# Acute Exacerbation of a Chronic Obstructive Pulmonary Disease Prediction System Using Wearable Device Data, Machine Learning, and Deep Learning: Development and Cohort Study

**DOI:** 10.2196/22591

**Published:** 2021-05-06

**Authors:** Chia-Tung Wu, Guo-Hung Li, Chun-Ta Huang, Yu-Chieh Cheng, Chi-Hsien Chen, Jung-Yien Chien, Ping-Hung Kuo, Lu-Cheng Kuo, Feipei Lai

**Affiliations:** 1 Department of Computer Science and Information Engineering National Taiwan University Taipei Taiwan; 2 Graduate Institute of Biomedical Electronics and Bioinformatics National Taiwan University Taipei Taiwan; 3 Department of Internal Medicine National Taiwan University Hospital, College of Medicine, National Taiwan University Taipei Taiwan; 4 Department of Environmental and Occupational Medicine National Taiwan University Hospital, College of Medicine, National Taiwan University Taipei Taiwan

**Keywords:** chronic obstructive pulmonary disease, clinical decision support systems, health risk assessment, wearable device

## Abstract

**Background:**

The World Health Organization has projected that by 2030, chronic obstructive pulmonary disease (COPD) will be the third-leading cause of mortality and the seventh-leading cause of morbidity worldwide. Acute exacerbations of chronic obstructive pulmonary disease (AECOPD) are associated with an accelerated decline in lung function, diminished quality of life, and higher mortality. Accurate early detection of acute exacerbations will enable early management and reduce mortality.

**Objective:**

The aim of this study was to develop a prediction system using lifestyle data, environmental factors, and patient symptoms for the early detection of AECOPD in the upcoming 7 days.

**Methods:**

This prospective study was performed at National Taiwan University Hospital. Patients with COPD that did not have a pacemaker and were not pregnant were invited for enrollment. Data on lifestyle, temperature, humidity, and fine particulate matter were collected using wearable devices (Fitbit Versa), a home air quality–sensing device (EDIMAX Airbox), and a smartphone app. AECOPD episodes were evaluated via standardized questionnaires. With these input features, we evaluated the prediction performance of machine learning models, including random forest, decision trees, k-nearest neighbor, linear discriminant analysis, and adaptive boosting, and a deep neural network model.

**Results:**

The continuous real-time monitoring of lifestyle and indoor environment factors was implemented by integrating home air quality–sensing devices, a smartphone app, and wearable devices. All data from 67 COPD patients were collected prospectively during a mean 4-month follow-up period, resulting in the detection of 25 AECOPD episodes. For 7-day AECOPD prediction, the proposed AECOPD predictive model achieved an accuracy of 92.1%, sensitivity of 94%, and specificity of 90.4%. Receiver operating characteristic curve analysis showed that the area under the curve of the model in predicting AECOPD was greater than 0.9. The most important variables in the model were daily steps walked, stairs climbed, and daily distance moved.

**Conclusions:**

Using wearable devices, home air quality–sensing devices, a smartphone app, and supervised prediction algorithms, we achieved excellent power to predict whether a patient would experience AECOPD within the upcoming 7 days. The AECOPD prediction system provided an effective way to collect lifestyle and environmental data, and yielded reliable predictions of future AECOPD events. Compared with previous studies, we have comprehensively improved the performance of the AECOPD prediction model by adding objective lifestyle and environmental data. This model could yield more accurate prediction results for COPD patients than using only questionnaire data.

## Introduction

With rapid progress of medicine, many treatments and medications have been developed, and relationships between lifestyle and disease have been elucidated. Precision medicine involves determining the best treatment plan for individual patients. Currently, research on precision medicine primarily involves developing related apps based on historical data from electronic medical records. When a patient is discharged from the hospital, lifestyle and environmental risks affect disease control. However, such factors are difficult to collect and use for analysis.

The World Health Organization includes chronic respiratory diseases among the four major human chronic diseases; in particular, lung disease accounts for an estimated 7.5 million deaths per year, or approximately 14% of annual deaths worldwide. These diseases are a major economic burden, and contribute to gender and social inequalities within and between countries. In descending frequency, the most frequent diseases include chronic obstructive pulmonary disease (COPD), lung cancer, tuberculosis, lung infections, asthma, and interstitial lung diseases [1]. COPD is a highly prevalent lung disease characterized by persistent airflow limitation due to a mixture of obstructive bronchiolitis and emphysema. The morbidity and mortality of COPD are high and continue to increase [2], such that COPD is projected to become the third-leading cause of death worldwide by 2030.

Acute exacerbation of COPD (AECOPD) decreases the patient’s quality of life, accelerates decline in lung function, and is significantly associated with mortality [3]. COPD is a heterogeneous disorder with large variations in the risk of exacerbation across patients. In clinical practice, a history of two or more exacerbations and one severe exacerbation per year is used to guide therapeutic choices for exacerbation prevention [3]. However, this approach is clinically limited owing to significant heterogeneity in risk even among those who have frequent exacerbation episodes. Although these outcomes may be avoided with early detection and treatment, increasing evidence shows that environmental and lifestyle factors may affect the development of COPD.

Lifestyle modification is considered to be one of the most cost-effective strategies in the self-management and secondary prevention of COPD [4]. Nevertheless, there is limited evidence demonstrating the relationship between lifestyle factors and COPD development. Several studies have developed predictive models for AECOPD [5]; however, there is no prediction tool incorporating both lifestyle data and medical questionnaires. Moreover, some researchers have argued that remote monitoring is a promising alternative—or an adjunct—to traditional health care services in COPD management [6]. Nonetheless, some studies have shown that inefficient systems, poor patient compliance, and poor performance of prediction tools may decrease the effects of health care interventions [7-9].

Hence, the objectives of this study were to (1) develop a lifestyle observation platform based on wearable devices and a smartphone app to observe lifestyle and environmental factors for patients with COPD, and to (2) construct an AECOPD prediction tool for the early prediction of COPD exacerbations using lifestyle factors, indoor environmental factors, and medical questionnaires.

## Methods

### Data Collection

Eligible participants were adult patients with COPD (20 years of age or older) who were not implanted with a pacemaker and who were not pregnant. Participants were recruited from the pulmonologist clinics at National Taiwan University Hospital between March 2019 and February 2020. The study protocol was approved by the institutional review board of National Taiwan University Hospital (201710066RINB). During the study period, we enrolled 67 patients with a confirmed diagnosis of COPD according to the Global Initiative for Chronic Obstructive Lung Disease (GOLD) criteria [3], as defined by the ratio of <70% postbronchodilator forced expiratory volume in 1 second to the forced vital capacity.

Data collection was based on clinical questionnaires, environmental data, and physiological data. Data on patient symptoms were prospectively evaluated by the modified Medical Research Council (mMRC) dyspnea scale and the COPD assessment test (CAT) upon enrollment and every month during follow-up. The mMRC scale is used to assess functional impairment due to dyspnea attributable to respiratory disease, and the CAT is a patient-completed questionnaire that is used globally to assess the impact of COPD (cough, sputum, dyspnea, chest tightness) on health status. Both are widely used clinical tests for COPD, and some studies [4,10] have also used these clinical tools to evaluate the health condition of COPD patients. According to the GOLD guidelines, COPD exacerbations are defined as the acute worsening of respiratory symptoms, resulting in additional therapy [11].

Environmental data and physiological data were collected as time-series data by home air quality–sensing devices and wearable devices that were provided to all participants. Although most medical research [4,10,12] tends to use medical devices as data sources, patient compliance could be reduced by the inconvenience and difficulty in operating such devices. Physiological data included walking steps, climbing stairs, distances, consumption in calories, heart rate, and sleep status. Environmental data comprised temperature, humidity, and fine particulate matter (PM2.5) levels. All data were synchronized with the database every 15 minutes to ensure that any subtle changes were not neglected.

### System Architecture

**Figure 1:**
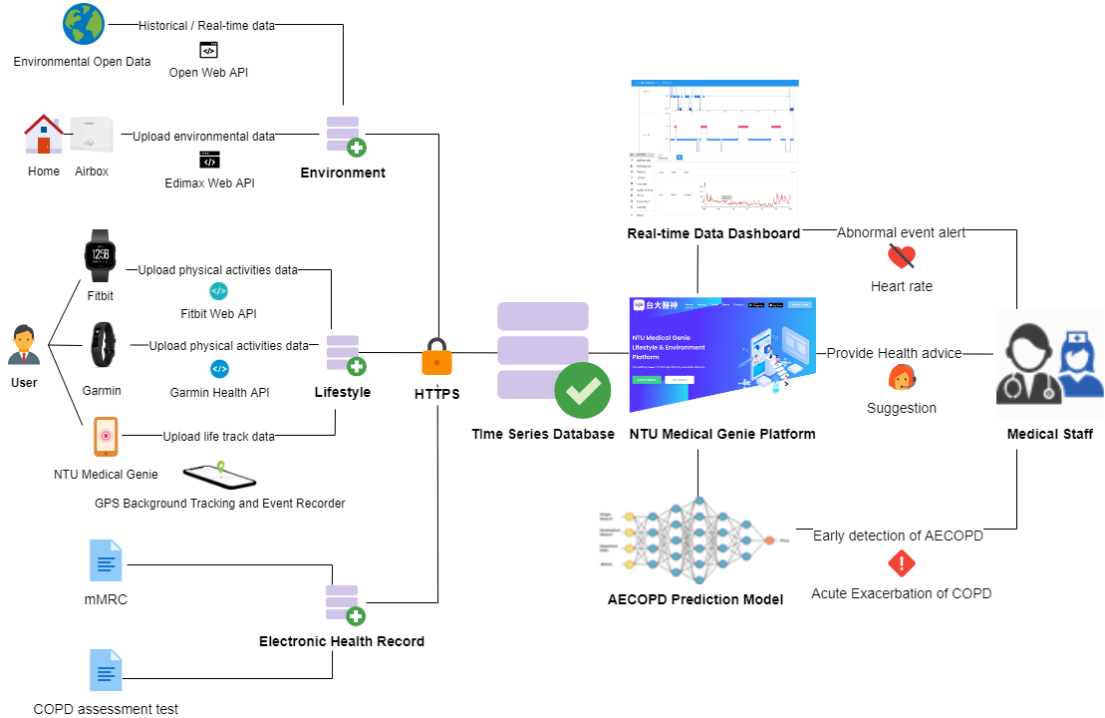
System architecture of the acute exacerbation of chronic obstructive pulmonary disease (AECOPD) prediction system. API: application programming interface; COPD: chronic obstructive pulmonary disease; mMRC: modified Medical Research Council dyspnea scale.

**Figure 2:**
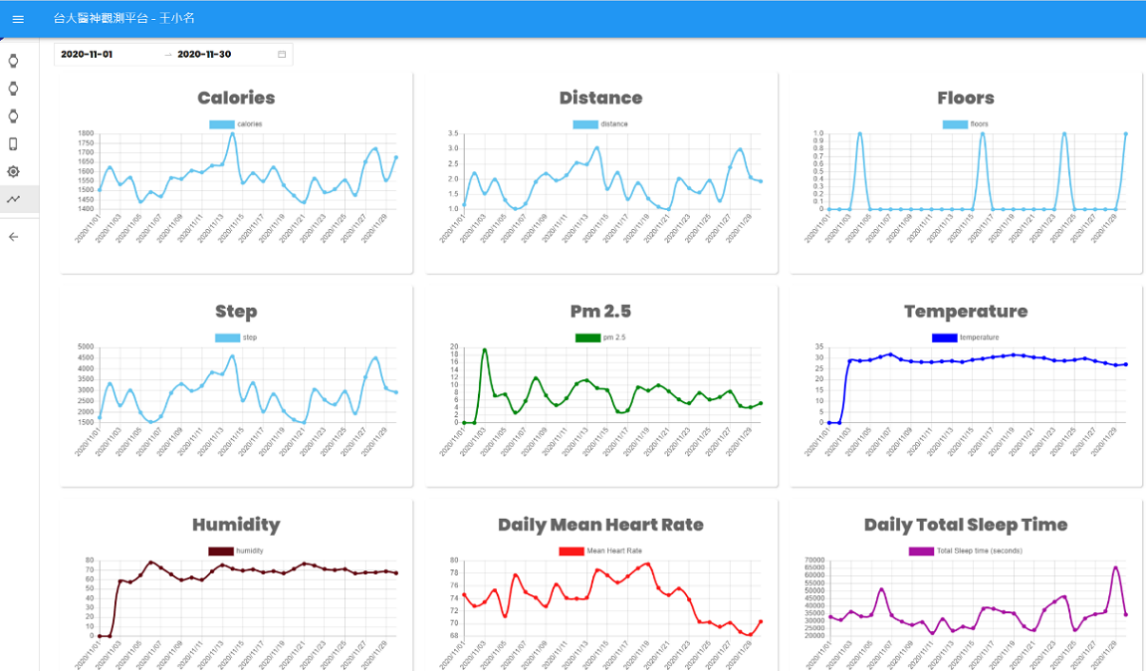
Data visualization from the lifestyle observation platform. Pm2.5: fine particulate matter.

Figure 1 shows the architecture of the AECOPD prediction system. The system consists of four components: a wearable device (Fitbit Versa), home air quality–sensing device (EDIMAX Airbox), lifestyle observation platform, and the personal health advice app. Wearable devices automatically collected lifestyle data (physical activities, heart rate, and sleep patterns) via Bluetooth to the original customized apps and were further connected to the lifestyle observation platform via the OAuth 2.0 protocol. Environmental data in the patient’s living environment (ie, temperature, humidity, and PM2.5 levels) were collected from the home air quality–sensing device and open environmental application programming interface (API). We also developed a health self-management smartphone app for patients and a lifestyle observation platform for medical staff, which together facilitate the continuous monitoring of lifestyle data and instant care advice. To assist physicians in organizing key information more effectively, data are visualized by combining trend charts, as shown in Figure 2. In addition to these trend charts, the daily prediction results are also used as a reference for decision support to help physicians better understand the status of their patients.

**Figure 3:**
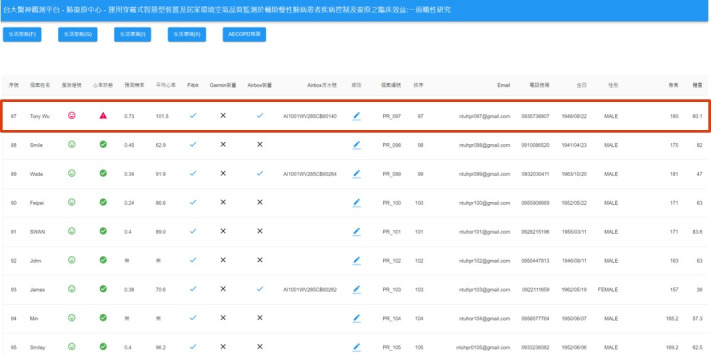
Daily prediction of acute exacerbation of chronic obstructive pulmonary disease.

**Figure 4:**
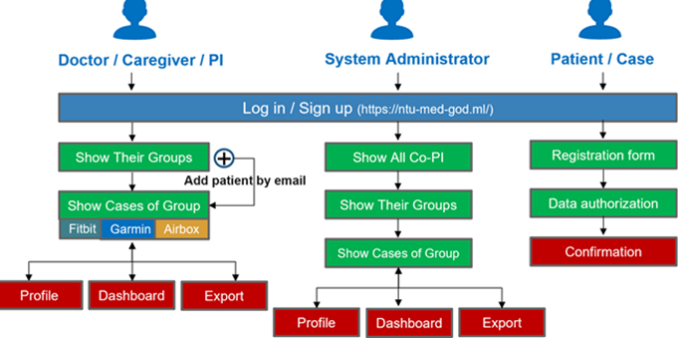
Data hierarchy workflow. PI: principal investigator.

**Figure 5:**
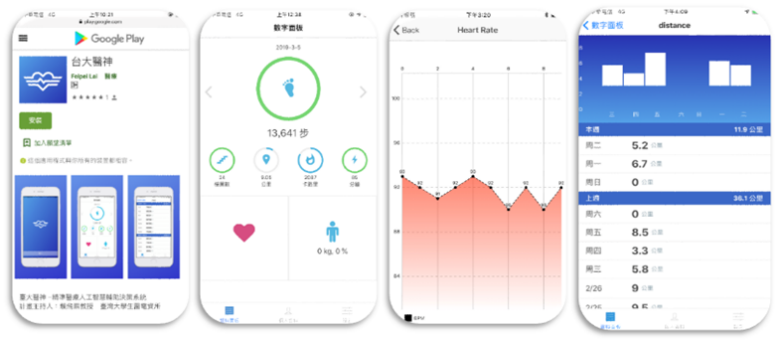
Screenshots of NTU-med-good health advice app.

Figure 3 shows an example system scenario: when the prediction probability exceeds 0.7, a red icon is displayed to prompt the case manager to intervene and take care of the patient. To protect patient privacy, the system transmits data via the HTTPS protocol, and personal information is encrypted. Figure 4 shows the data management workflow. Only verified medical service providers can access their patients’ information, which ensures the confidentiality and integrity of the data. The mobile app was designed to record symptoms and produce trend charts to help patients better understand their health status, as shown in Figure 5. With appropriate expert advice, they are thus able to better manage their own health.

### Data Processing

**Figure 6:**
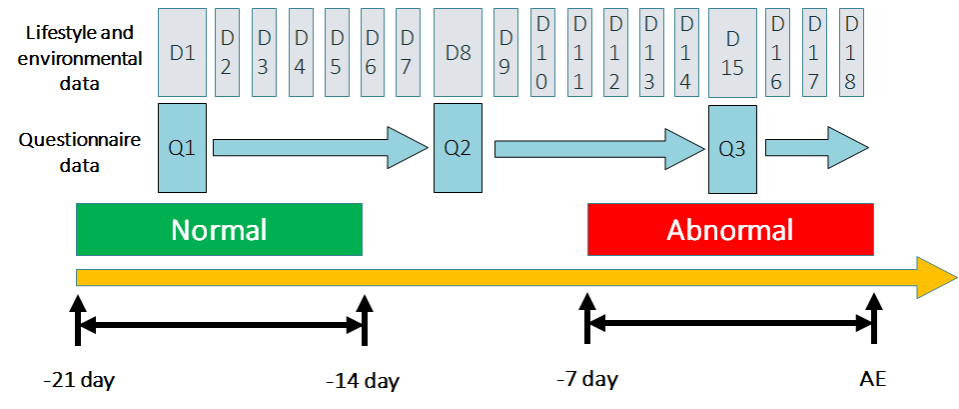
Forward padding for questionnaire data. AE: acute exacerbation.

**Figure 7:**
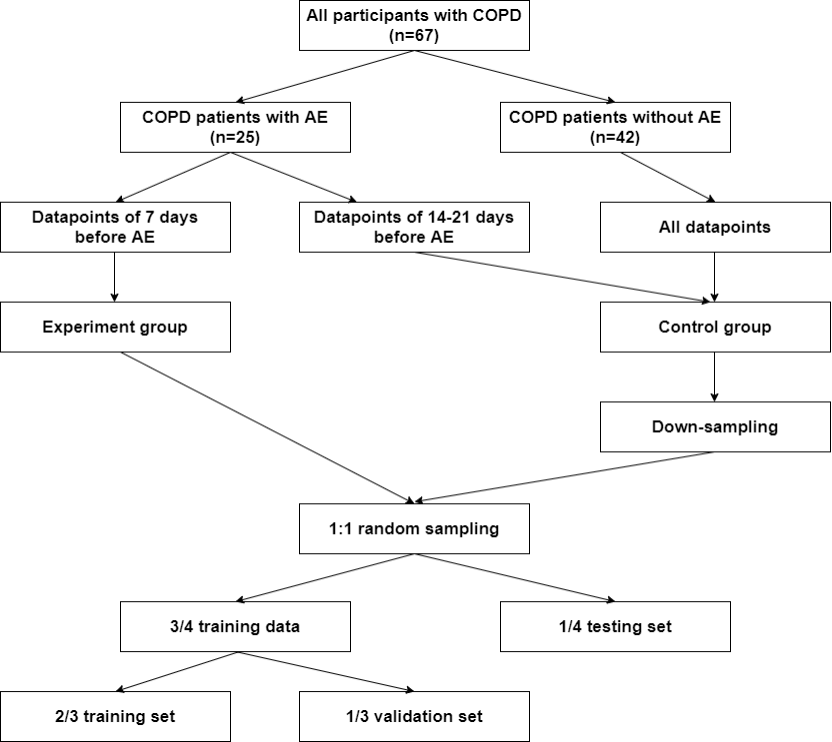
Decision rules for data selection. COPD: chronic obstructive pulmonary disease; AE: acute exacerbation.

**Figure 8:**
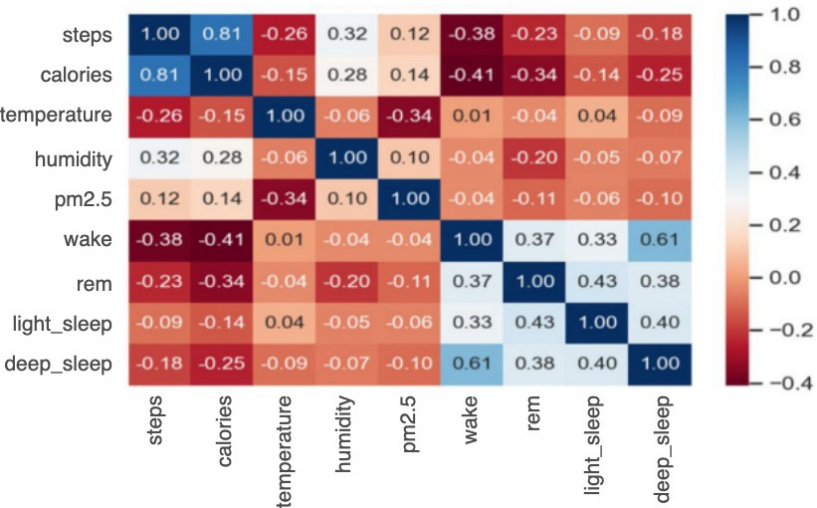
Correlation matrix of physiological and environmental data.

The combination of clinical questionnaire data, environmental data, and physiological data was the main dataset used in our training model. To explore the influence of changes in environmental and lifestyle factors on AECOPD, first- and second-order differentiation models were applied to the environmental and physiological data to understand trends and serve as additional input features. Random downsampling was used to account for data imbalance, resulting in 5600 data points, one-third of which were used as the validation set and the remainder were used as the training set. We used forward-filling to pad missing and questionnaire data, as illustrated in Figure 6. The complete data selection rules are shown in Figure 7. Note that missing values is a common problem in data mining. A correlation analysis (Figure 8) was performed between physiological and environmental features to ensure that their interactions did not affect the prediction results. Finally, as shown in Textbox 1, 45 features were selected to predict the probability of AECOPD.

**Input data features of machine-learning and deep-learning models.**

- Environmental factors

Temperature, humidity, fine particulate matter, first-order differentiation (diff1)_temperature, diff1_humidity, diff1_fine particulate matter, second-order differentiation (diff2)_temperature, diff2_humidity, diff2_fine particulate

- Physiological factors

Heart rate, walking steps, calories consumption, deep sleep time, light sleep time, rapid eye movement time, awake time, diff1_heart rate, diff1_ walking steps, diff1_calories consumption, diff1_deep sleep time, diff1_light sleep time, diff1_rapid eye movement time, diff1_awake time, diff2_heart rate, diff2_ walking steps, diff2_calories consumption, diff2_deep sleep time, diff2_light sleep time, diff2_rapid eye movement time, diff2_awake time

- Clinical questionnaires

Chronic obstructive pulmonary disease (COPD) assessment test (9 answers), modified Medical Research Council (mMRC) dyspnea scale (1 answer), life quality questionnaire (5 answers)

### Classification Models

Classification algorithms for this study were selected according to previously published studies on COPD such as those of Wang et al [13] and Rahman et al [14]. The former group developed AECOPD identification models to reduce patient mortality and financial burdens, and the latter group attempted to identify relations between discriminatory heart rate variability features and disease severity in patients with pulmonary diseases and COPD. For model comparison with machine learning–based classification, we selected the following classifiers: decision trees [15], random forests [16], k-nearest neighbor clustering [17], linear discriminant analysis, and adaptive boosting [18]. We also propose a deep neural network (DNN) architecture for use in comparing the performance between machine-learning and deep-learning approaches on AECOPD prediction. Supervised learning was performed using AECOPD events and 51 features obtained from the lifestyle observation platform. Models were implemented using python libraries such as scikit-learn and Pytorch.

### DNN Classification

**Figure 9:**
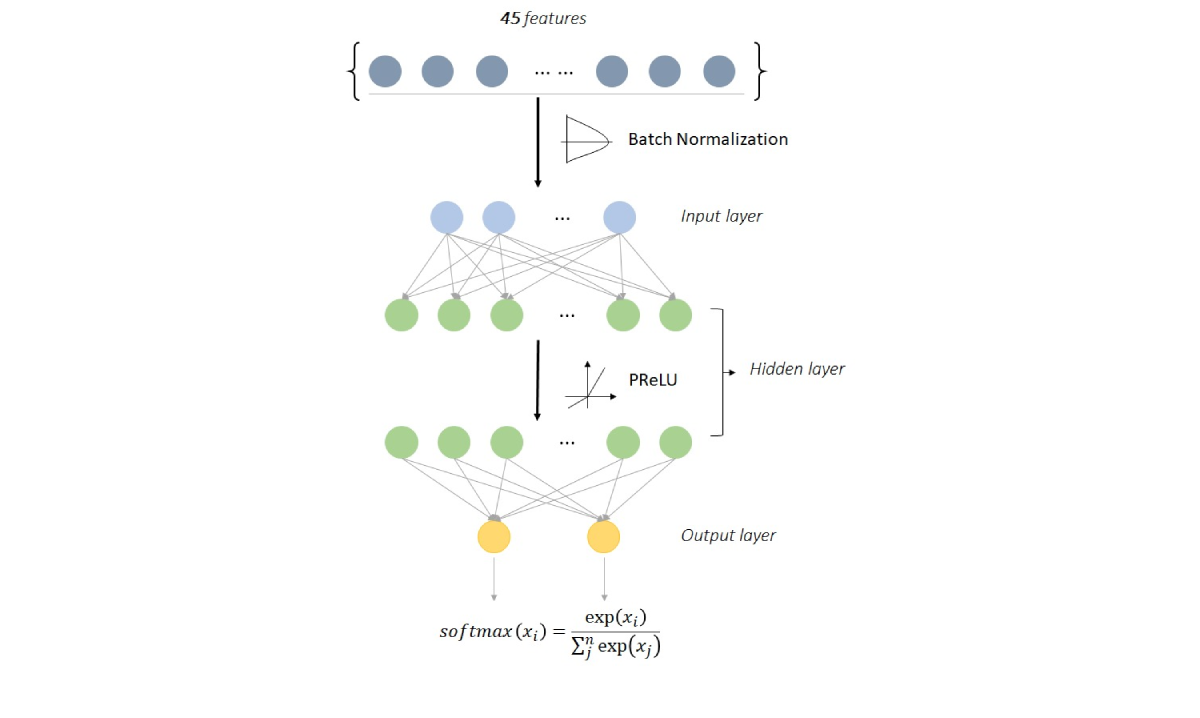
Deep neural network model architecture. PReLU: parametric rectified linear unit.

The DNN classification model was constructed using fully connected layers, which connect each neuron in one layer to every neuron in another layer, mapping feature representations to the target vector space. Hyperparameters for the two fully connected layers are presented in Table 1. Batch normalization was applied to input data sequences to reduce the internal covariate shift and gradient dependence [19]. For the activation function we used parametric rectified linear unit (PReLU), which combines the characteristics of ReLU and leaky ReLU, with the introduction of a variable slope α, randomly selected from the uniform distribution during the training process to negative values [20]. The complete DNN architecture is shown in Figure 9.

**Table 1:** Hyperparameters of the machine-learning and deep-learning models.

Model and hyperparameters		Value
**Decision trees**		
	min_samples_leaf	1
	min_samples_split	2
AdaBoost: n_estimators		45
**Random forests**		
	min_samples_leaf	1
	min_samples_split	2
	n_estimators	100
**k-nearest neighbor**		
	n_neighbors	3
	leaf_size	30
	p	2
**Deep neural network**		
	fully connected layer 1	45
	fully connected layer 2	45

### Validation and Model Assessment

**Figure 10:**
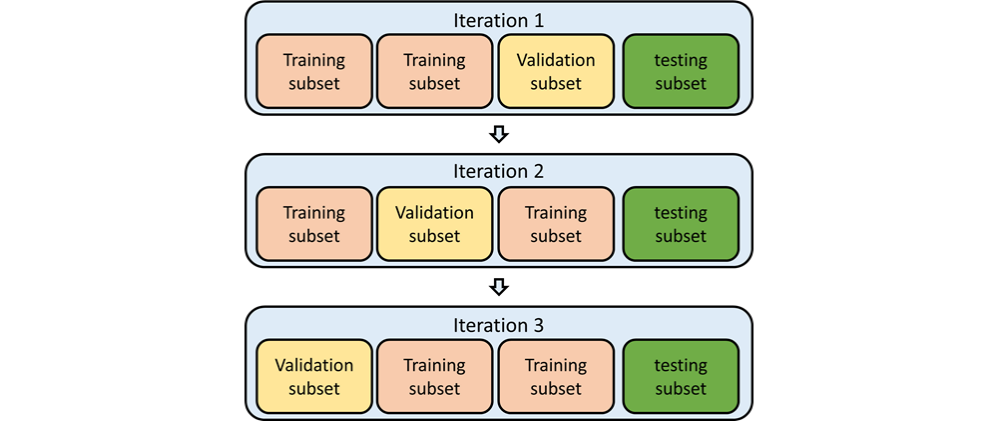
Workflow of 3-fold cross-validation.

We use 3-fold cross-validation to evaluate the stability of the prediction models. The workflow is shown in Figure 10. We used two metrics to evaluate the performance of the identification models based on the test set: the area under the receiver operating characteristic curve (AUROC) and the F1 score. We also used sensitivity, specificity, precision, and accuracy as assessment metrics.

## Results

### Patient Characteristics

A total of 67 patients were registered for this study. Most of the patients were middle-aged (mean 66.62, SD 11.38 years) and were men. Eighteen percent had never smoked and the remainder were either current smokers or exsmokers. Detailed demographic information of the study participants is shown in Table 2.

**Table 2:** Demographics of study participants (N=67).

Characteristic		Value
Age (years), mean (SD)		66.62 (11.38)
**Gender, n (%)**		
	Male	59 (88)
	Female	8 (12)
**Smoking history, n (%)**		
	Never smoker	18 (27)
	Current smoker	9 (13)
	Exsmoker	40 (60)
**Comorbidities**		
	Diabetes mellitus	12 (18)
	Hypertension	25 (37)
	Myocardial infarction	1 (1)
	Heart failure	2 (3)
	Peripheral vascular disease	11 (16)
	Bronchiectasis	15 (22)
	Postnasal drip syndrome	6 (9)
	Nasal septum deviation	5 (7)
	Allergic rhinitis	19 (28)
	Others	24 (36)
**FEV1^a^(% predicted), n (%)**		
	≥80	14 (21)
	50-79	24 (36)
	30-49	20 (30)
	<30	9 (13)

^a^FEV1: postbronchodilator forced expiratory volume in 1 second.

### Distribution of Physiological and Environmental Factors

**Figure 11:**
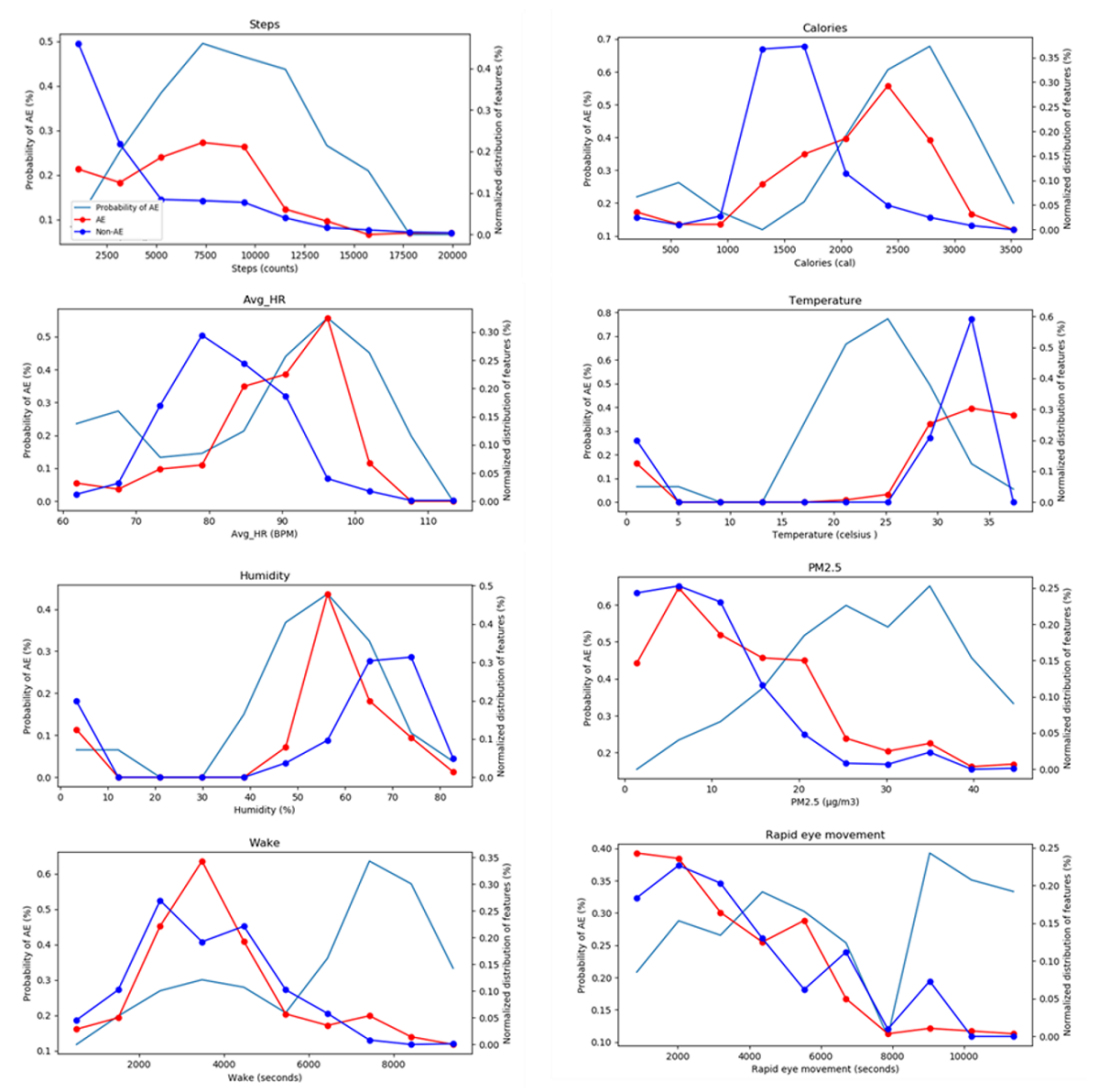
Acute exacerbation of chronic obstructive pulmonary disorder probability trends versus normalized distributions of physiological and environmental factors. HR: heart rate; AE: acute exacerbation: PM2.5: fine particulate matter.

Figure 11 illustrates the AECOPD probabilities versus the distributions of physiological and environmental features, among which average heart rate, PM2.5, steps walked, and calorie consumption were significantly different between those with and without AECOPD. This shows that physiological and environmental factors are useful for predicting AECOPD.

### AECOPD Prediction Model

**Figure 12:**
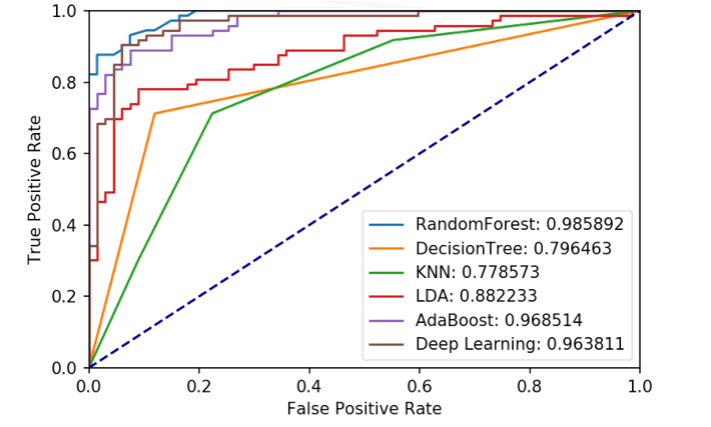
Receiver operating characteristic (ROC) curve and area under the receiver operating characteristic curve of all models. AE: acute exacerbation; KNN: k-nearest neighbor; LDA: linear discriminant analysis.

Table 3 and Figure 12 demonstrate the performance of the implemented models. The DNN model yielded the best performance with 6 metrics higher than 90%.

To determine which model best fits diverse scenarios, we trained the model using various combinations of data features, as shown in Table 4: the prediction including all of the features yielded the best performance. These results further confirmed that physiological and environmental data features are more predictive of AECOPD than conventional clinical questionnaires.

**Table 3:** Performance of each model with all features input.

Model	Accuracy	AUCROC^a^	Sensitivity	Specificity	Precision	F1
Random forests	0.914	0.986	0.877	0.955	0.955	0.914
Decision trees	0.792	0.796	0.712	0.881	0.867	0.782
kNN^b^	0.743	0.779	0.712	0.776	0.776	0.743
LDA^c^	0.829	0.882	0.781	0.881	0.877	0.826
AdaBoost^d^	0.886	0.969	0.822	0.955	0.952	0.882
DNN^e^	0.921	0.964	0.904	0.940	0.943	0.923

^a^AUROC: area under the receiver operating characteristic curve.

^b^kNN: k-nearest neighbor.

^c^LDA: linear discriminant analysis.

^d^AdaBoost: adaptive boosting.

^e^DNN: deep neural network.

**Table 4:** Performance given different feature sets.

Feature	Model	Accuracy	AUROC^a^	Sensitivity	Specificity	Precision	F1
All features	DNN^b^	0.9357	0.9699	0.9452	0.9253	0.9393	0.9323
Lifestyle	Random forests	0.8428	0.9195	0.9253	0.7671	0.9180	0.8358
Env^c^	Decision trees	0.8000	0.8185	0.8805	0.7260	0.8688	0.7910
Env+Lifestyle	Random forests	0.8357	0.9256	0.8805	0.7945	0.8787	0.8345
Clinical questionnaire	AdaBoost^d^	0.6956	0.6825	0.6666	0.7142	0.7692	0.7407

^a^AUROC: area under the receiver operating characteristic curve.

^b^DNN: deep neural network.

^c^Env: environmental.

^d^AdaBoost: adaptive boosting.

### AECOPD Prediction System

**Figure 13:**
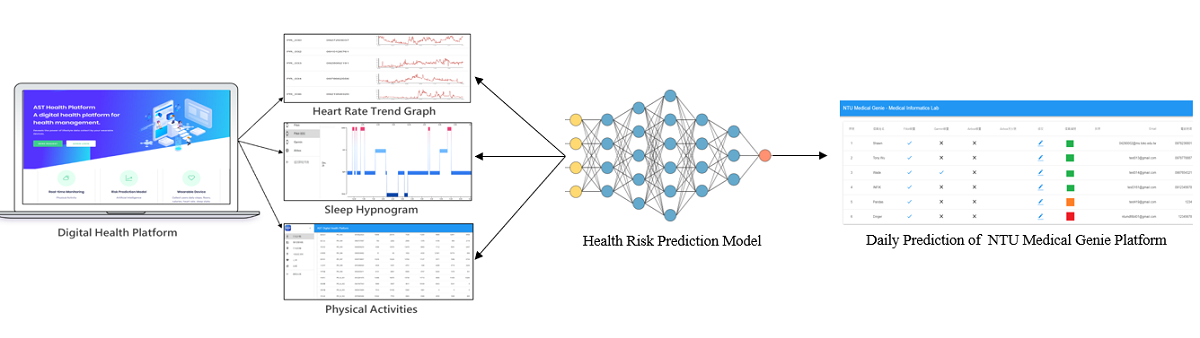
Acute exacerbation of chronic obstructive pulmonary disease prediction system.

To account for incomplete data, which is typical in real-world apps, the prediction system supports AECOPD prediction via optional features. When only lifestyle or environmental data are automatically uploaded daily, the system still predicts whether AECOPD will occur within the next 7 days. Therefore, multiple AECOPD prediction models were deployed on the server. Through the process shown in Figure 13, daily prediction results are provided to support physicians in making decisions. Different color signs displayed on the system indicate different risk levels.

### Feature Importance

**Figure 14:**
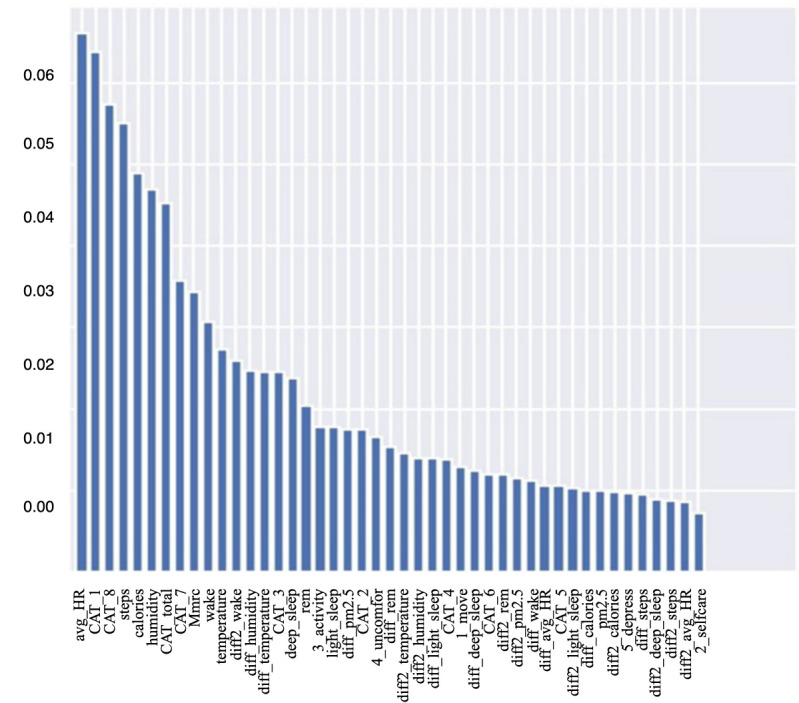
Feature importance scores as evaluated by random forest.

Figure 14 shows the importance scores of model features as evaluated by a random forest algorithm. Feature importance is a measure of the ability to improve the purity of the random forest model’s leaf nodes. Daily activity–related features such as average heart rate, calorie consumption, and steps walked yielded higher importance scores, which indicates that these features have greater potential to improve the performance of the random forest model. As average heart rate had the highest importance score, and is thus likely the most influential predictor of AECOPD, we used a warning sign for abnormal heart rates in the AECOPD prediction system to alert physicians when the patient’s average heart rate was abnormal.

## Discussion

### Principal Findings

We implemented an AECOPD prediction system by integrating wearable devices, Internet-of-Things environment sensors, a smartphone app, machine learning, and deep learning. We present six models for AECOPD identification. Additionally, we selected features to determine the optimal feature set for this task. The performance of each model is demonstrated according to sensitivity, specificity, F1-score, accuracy, precision, and AUROC metrics obtained based on 3-fold cross-validation. To the best of our knowledge, this is the most comprehensive study that used machine-learning models to predict AECOPDs.

Clinical questionnaires tend to be more subjective, which can affect clinical decisions. The AECOPD prediction model with all data features achieved the best performance. These results showed that physiological and environmental data are more powerful predictors than questionnaire data. Compared with clinical questionnaire data alone, lifestyle and environmental data yielded improvements of 10% in accuracy and 20% in AUROC.

### Comparison With Prior Work

In the 2010s, researchers began to attempt to predict COPD exacerbation. One study used demographic features, vital signs, and electronic medical records to predict COPD exacerbations in the emergency department [21]. Another used 28 features, including vital signs, medical history, inflammatory indicators, and tree-based machine learning, to predict the prognosis of hospitalized patients with COPD [22]. In contrast, in this study, we focused on exacerbation risk prediction for discharged COPD patients, because their health condition is likely to be less accessible. Another study remotely monitored AECOPD in patients via questionnaire data. They demonstrated an accuracy of 100% for event-based prediction and up to 80.5% for symptom-based prediction [23]. In addition, Shah et al [24] used pulse oximetry and three vital signs to predict AECOPD, reaching a mean AUROC of 68%. In comparison to these studies, we used daily activities and environmental information as predictors to trace the health conditions of patients with COPD and achieved higher performance. With wearable devices and smartphone apps, all relevant COPD information can be collected instantly. Such a system will be helpful for achieving the goal of personalized health management in the future. Thus, overall, our study constitutes a novel solution making use of various data sources for superior AECOPD prediction performance.

### Limitations

Because of limitations in the air quality–sensing device, environmental data collection was restricted to the user’s bedroom, which degrades the prediction results. To account for this, in the future we plan to use GPS functions to trace the user’s movements to capture the corresponding environmental data published via governmental open APIs.

In contrast to assumptions of physicians, Figure 10 shows that patients with AECOPD engaged in more physical activity than those without AECOPD. After reviewing the personal lifestyle of each patient, we found that some engaged in intense exercise even if they were uncomfortable, which goes against the accepted knowledge of the medical profession. In the future, more data could help to shed light on this apparent paradox.

### Conclusions

Patients with COPD generally must return to the hospital monthly for numerous clinical tests, which is a time-consuming procedure. At the same time, it is impractical for discharged patients or those under home care to continuously observe their health conditions. They thus run the risk of AECOPDs between routine visits.

In this study, we attempted to predict whether a patient with COPD will experience acute exacerbation of their condition within the next 7 days. In general, lifestyle and environmental data of patients are difficult to collect effectively. However, with the proposed system, all COPD-related data are uploaded automatically. Our results indicate that lifestyle and environmental data facilitate the precise management of users’ health conditions, and can even produce early warnings of AECOPD. The experimental results confirmed that such lifestyle and environmental data are highly correlated to user health conditions. In the future, we will enhance the prediction system and perform external validation to ensure that the model can be applied to other regions.

## Abbreviations

AECOPD: acute exacerbation of chronic obstructive pulmonary disease
API: application programming interface
AUROC: area under the receiver operating characteristic curve
CAT: chronic obstructive pulmonary disease assessment test
COPD: chronic obstructive pulmonary disease
DNN: deep neural network
GOLD: Global initiative for chronic obstructive lung disease
mMRC: modified Medical Research Council dyspnea scale
PM2.5: fine particulate matter
